# Ferroptosis in Anaplastic Thyroid Cancer: Molecular Mechanisms, Preclinical Evidence, and Therapeutic Prospects

**DOI:** 10.3390/cells14221800

**Published:** 2025-11-17

**Authors:** Jaewang Lee, Jong-Lyel Roh

**Affiliations:** 1 Department of Otorhinolaryngology-Head and Neck Surgery, CHA Bundang Medical Center, CHA University, Seongnam 13496, Republic of Korea; 2 Logsynk, Seoul 11160, Republic of Korea; 3 Department of Biomedical Science, General Graduate School, CHA University, Pocheon 11160, Republic of Korea

**Keywords:** anaplastic thyroid cancer, ferroptosis, lipid peroxidation, targeted therapy, biomarkers

## Abstract

**Highlights:**

**What are the main findings?**

- ATC exhibits ferroptosis vulnerability due to dysregulation of iron and lipid metabolism.
- Genetic regulators, including SIRT6, EIF3H–β-catenin, and GPR34–USP8, shape ferroptosis sensitivity.
- RON signaling links glycolysis to ferroptosis resistance, offering a new therapeutic target.

**What are the implications of the main findings?**

- Natural compounds such as vitamin C, neferine, curcumin, and shikonin induce ferroptosis in ATC.
- Anlotinib triggers ferroptosis via ROS and ER stress, amplified by autophagy blockade.
- Combination regimens, including BRAF inhibitors with GPX4 blockade or isobavachalcone plus doxorubicin, enhance ATC suppression.

**Abstract:**

Anaplastic thyroid cancer (ATC) is among the most lethal human malignancies, characterized by rapid progression, therapeutic resistance, and a median survival of less than one year. Conventional therapies, including surgery, radiotherapy, and chemotherapy, have limited effect, and targeted or immune-based treatments provide only transient benefit. Ferroptosis, a regulated form of cell death driven by iron-dependent lipid peroxidation, has recently emerged as a therapeutic vulnerability in ATC. This review synthesizes current evidence on ferroptosis biology, preclinical validation, and therapeutic implications in ATC. Genomic alterations such as TP53, BRAF^V600E^, RAS, and PIK3CA converge on redox imbalance and metabolic rewiring, rendering ATC cells dependent on antioxidant defenses. Dysregulated iron homeostasis through ferritinophagy and HO-1 activity, together with lipid remodeling via ACSL4 and LPCAT3, further sensitizes ATC to ferroptosis. Preclinical studies show that pharmacological inducers, including vitamin C, tenacissoside H, neferine, curcumin, and shikonin, as well as targeted agents such as dabrafenib and anlotinib, can trigger or synergize with ferroptosis. Genetic regulators, including SIRT6, the GPR34–USP8 axis, and the EIF3H–β-catenin pathway, modulate ferroptosis sensitivity, while RON receptor signaling links glycolysis to ferroptosis resistance. Combination regimens provide further translational potential. Nanoplatforms also offer innovative delivery strategies. Therapeutic approaches include initiating ferroptosis through iron and PUFA enrichment, disabling defenses such as GPX4 and Nrf2, and integrating ferroptosis inducers with existing modalities. Although systemic toxicity and resistance remain obstacles, biomarker-driven selection and drug repurposing offer promise. Ferroptosis represents a mechanistically distinct and clinically exploitable pathway for ATC.

## 1. Introduction

Anaplastic thyroid cancer (ATC) is among the most aggressive solid malignancies, representing only 1–2% of thyroid cancers but accounting for up to 50% of thyroid cancer-related deaths worldwide [1]. Patients often present with rapidly enlarging neck masses, local invasion, and distant metastases, and the median overall survival remains less than one year despite aggressive multimodal treatment [2]. Conventional therapies such as surgery, radiotherapy, and chemotherapy rarely achieve durable control, and even the integration of multikinase inhibitors or BRAF/MEK-targeted therapy has provided only incremental benefits, with resistance and relapse being nearly universal [3]. These sobering outcomes underscore the urgent need for novel therapeutic paradigms that can exploit unique vulnerabilities of ATC cells.

**Figure 1:**
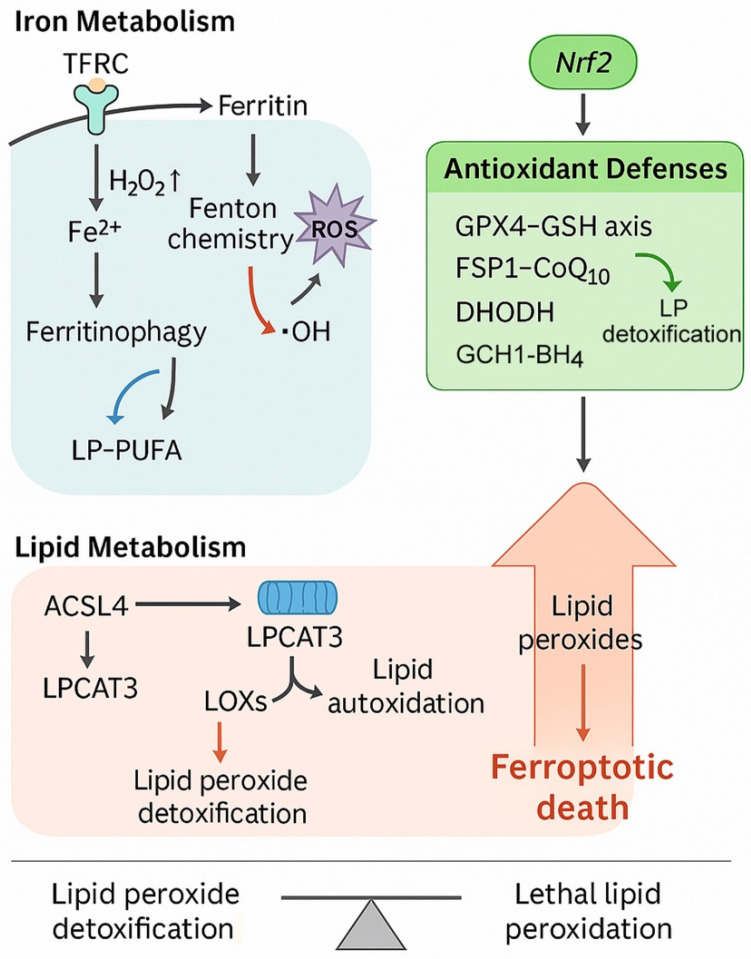
Core pathways orchestrating ferroptosis. Iron metabolism contributes to ferroptosis through transferrin receptor-mediated iron uptake (TFRC), ferritin degradation via ferritinophagy, and Fenton chemistry-driven production of reactive oxygen species (ROS). Excess ferrous iron (Fe^2+^) promotes hydroxyl radical (•OH) generation and lipid peroxidation of polyunsaturated fatty acids within phospholipids (LP-PUFA). Lipid metabolism regulates susceptibility to ferroptosis through ACSL4-mediated activation of polyunsaturated fatty acids and LPCAT3-dependent phospholipid remodeling. Lipoxygenases (LOXs) and lipid autoxidation further contribute to lipid peroxide accumulation. Antioxidant defenses suppress ferroptosis via multiple pathways, including the glutathione peroxidase 4–glutathione axis (GPX4–GSH), ferroptosis suppressor protein 1–coenzyme Q10 system (FSP1–CoQ_10_), dihydroorotate dehydrogenase (DHODH), and the GTP cyclohydrolase 1–tetrahydrobiopterin axis (GCH1–BH4). Nrf2 transcriptionally upregulates these detoxifying systems. Abbreviations: ACSL4, acyl-CoA synthetase long-chain family member 4; BH4, tetrahydrobiopterin; CoQ_10_, coenzyme Q10; DHODH, dihydroorotate dehydrogenase; Fe^2+^, ferrous iron; FSP1, ferroptosis suppressor protein 1; GCH1, GTP cyclohydrolase 1; GPX4, glutathione peroxidase 4; GSH, glutathione; LPCAT3, lysophosphatidylcholine acyltransferase 3; LOXs, lipoxygenases; LP-PUFA, polyunsaturated fatty acid-containing phospholipids; Nrf2, nuclear factor erythroid 2-related factor 2; ROS, reactive oxygen species; TFRC, transferrin receptor; •OH, hydroxyl radical. The biological rationale for investigating ferroptosis in ATC is compelling. ATC is genetically characterized by frequent mutations in TP53, TERT promoter, BRAF, and RAS, as well as alterations in PI3K/AKT and SWI/SNF chromatin remodeling complexes [8,9]. Many of these aberrations converge on redox imbalance, metabolic rewiring, and iron homeostasis, rendering ATC cells potentially vulnerable to ferroptosis. For instance, RAS mutations, present in 20–40% of ATCs, are known to sensitize cells to ferroptosis inducers, while p53 loss can disrupt cystine uptake regulation via SLC7A11, further enhancing susceptibility [10]. Similarly, dedifferentiated phenotypes and epithelial-to-mesenchymal transition (EMT), hallmarks of ATC progression, are associated with altered lipid metabolism and increased dependency on antioxidant defenses, providing additional entry points for ferroptosis-based interventions [11].

Ferroptosis, first described in 2012, is a distinct form of regulated cell death characterized by the iron-dependent accumulation of lethal lipid peroxides, setting it apart from apoptosis, necroptosis, and autophagy [4,5]. Its hallmarks include mitochondrial shrinkage with condensed membranes, loss of cristae, and catastrophic lipid peroxidation driven by polyunsaturated fatty acids (PUFAs) [6]. The process is tightly regulated by antioxidant defense systems, including glutathione peroxidase 4 (GPX4), ferroptosis suppressor protein 1 (FSP1), dihydroorotate dehydrogenase (DHODH), and the GTP cyclohydrolase 1 (GCH1)–tetrahydrobiopterin (BH_4_) axis [7]. Disruption of these defense pathways or excessive accumulation of ferrous iron can push cells beyond the oxidative threshold, culminating in ferroptotic cell death (Figure 1).

Recent preclinical studies have begun to substantiate this vulnerability. Anlotinib, a multikinase antiangiogenic inhibitor, was shown to suppress ATC cell proliferation and metastasis by activating the autophagy–ferroptosis axis and downregulating GPX4, ferritin heavy chain (FTH1), and heme oxygenase-1 (HO-1), while protective autophagy blockade further amplified ferroptosis and enhanced tumor regression [12]. Vitamin C at pharmacological doses induced ferritinophagy, iron accumulation, and reactive oxygen species (ROS) production, effectively triggering ferroptosis in ATC cells and suppressing long-term tumor growth [13]. In addition, natural compounds such as neferine and curcumin have demonstrated ferroptosis-inducing effects in ATC models by suppressing the Nrf2/HO-1 signaling pathway, linking phytochemicals with ferroptotic modulation [14]. Novel nanoplatforms, including Fe/curcumin-loaded ultrasound-responsive carriers, have also been designed to achieve targeted “domino-ferroptosis” in ATC xenografts, underscoring the translational momentum in this field [15].

Collectively, these findings suggest that ferroptosis represents a promising therapeutic vulnerability in ATC, with potential to synergize with existing targeted agents, immunotherapies, and radiotherapy [16]. Yet, several challenges remain, including the identification of predictive biomarkers (e.g., GPX4, SLC7A11, ACSL4, and FSP1 expression), the management of systemic toxicity, and the development of resistance mechanisms such as Nrf2 hyperactivation or monounsaturated fatty acid (MUFA) remodeling [17,18]. Addressing these barriers will be critical for translating ferroptosis-based strategies into clinical benefit for ATC patients.

The present review aims to systematically synthesize current evidence on ferroptosis in ATC, contextualized within the broader landscape of thyroid cancer biology. We will first outline the molecular framework of ferroptosis and its regulatory networks, then examine experimental evidence linking ferroptosis to ATC progression and therapy. We further discuss therapeutic strategies that exploit ferroptosis vulnerabilities, including small molecules, natural compounds, and combination regimens, and explore the roles of the tumor microenvironment and immune modulation. Finally, we highlight emerging biomarkers, ongoing translational efforts, and future perspectives for integrating ferroptosis into the treatment armamentarium against ATC.

## 2. Molecular Background and Ferroptosis Vulnerabilities in ATC

**Figure 2:**
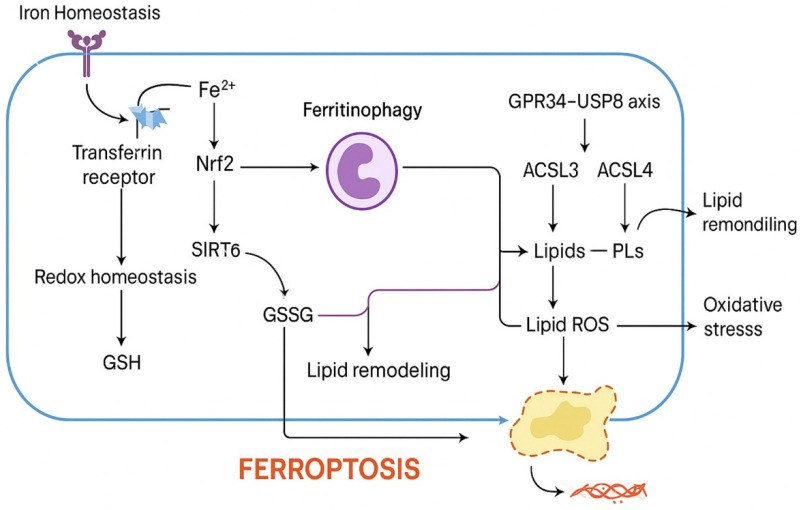
Ferroptosis-regulatory networks in anaplastic thyroid cancer (ATC). This schematic summarizes the major molecular regulators of ferroptosis in ATC by integrating iron homeostasis, redox balance, and lipid metabolism. Iron homeostasis is governed by transferrin receptor-mediated uptake of Fe^2+^ and subsequent redox regulation through GSH synthesis. Fe^2+^ accumulation activates Nrf2, which modulates antioxidant defenses, whereas SIRT6 promotes ferritinophagy and influences GSH/GSSG redox cycling. Lipid metabolism involves ACSL4-dependent incorporation of polyunsaturated fatty acids (PUFAs) into phospholipids, generating peroxidation-prone PUFA-PLs, while ACSL3 contributes to lipid remodeling through monounsaturated lipid synthesis. Ferritinophagy-derived iron and lipid remodeling converge to generate lipid ROS, leading to oxidative stress and ferroptotic cell death. The GPR34–USP8 axis reduces lipid ROS accumulation and represents a key anti-ferroptotic pathway. In this schematic, pro-ferroptotic factors include Fe^2+^ accumulation, ferritinophagy, SIRT6 activation, ACSL4-mediated PUFA-PL synthesis, lipid ROS generation, and oxidative stress, while anti-ferroptotic factors include Nrf2 signaling, GSH/GSSG antioxidant buffering, ACSL3-dependent lipid remodeling, and the GPR34–USP8 axis. Together, these interactions define the ferroptotic vulnerability and resistance mechanisms characteristic of ATC biology. Abbreviations: ACSL3, acyl-CoA synthetase long-chain family member 3; ACSL4, acyl-CoA synthetase long-chain family member 4; Fe^2+^, ferrous iron; GPR34, G-protein-coupled receptor 34; GSH, reduced glutathione; GSSG, oxidized glutathione; Nrf2, nuclear factor erythroid 2-related factor 2; PLs, phospholipids; PUFA-PLs, polyunsaturated fatty acid-containing phospholipids; ROS, reactive oxygen species; SIRT6, sirtuin 6; USP8, ubiquitin-specific protease 8.

ATC is distinguished from differentiated thyroid carcinomas not only by its aggressive clinical course but also by its distinct molecular landscape and metabolic features [19]. These characteristics contribute to unique vulnerabilities that intersect with ferroptosis, a regulated cell death process driven by iron-dependent lipid peroxidation. In this section, we outline the genetic, metabolic, and redox contexts that predispose ATC to ferroptosis while simultaneously enabling resistance, providing a framework for therapeutic exploitation (Figure 2).

### 2.1. Genomic Landscape and Ferroptosis Sensitivity

The genetic underpinnings of ATC are central to its biological behavior and therapeutic resistance. Comprehensive sequencing studies have revealed that ATC frequently harbors mutations in TP53 (>70%), TERT promoter (>70%), BRAF^V600E^ (~40%), and RAS (~20%), in addition to alterations in PI3K/AKT/mTOR signaling and the SWI/SNF chromatin remodeling complex [8,20]. Each of these aberrations influences redox balance, lipid metabolism, or iron handling, thereby shaping ferroptosis susceptibility.

Loss of TP53 is particularly relevant, as wild-type p53 represses the expression of the cystine transporter SLC7A11, thereby limiting cystine uptake and reducing glutathione (GSH) synthesis [21,22]. When TP53 is mutated, this regulation is lost, leading to increased antioxidant buffering and relative resistance to ferroptosis [23,24]. However, paradoxically, certain TP53 mutations may also increase metabolic stress and oxidative burden, thereby sensitizing ATC cells to ferroptosis under specific contexts [25,26]. Similarly, RAS mutations, found in both poorly differentiated thyroid cancer (PDTC) and ATC, elevate mitochondrial ROS and remodel lipid metabolism, rendering cells dependent on antioxidant defenses and more vulnerable to ferroptosis inducers [27]. BRAF^V600E^, although initially targetable with BRAF inhibitors, has been associated with adaptive resistance mediated through metabolic rewiring. Preclinical studies have shown that BRAF inhibition combined with ferroptosis induction can overcome this resistance, linking BRAF^V600E^-driven ATC directly to ferroptosis-based therapeutic strategies [28].

In addition to these canonical alterations, PIK3CA mutations are present in a subset of ATCs and contribute to ferroptosis regulation. Hyperactivation of PI3K/AKT/mTOR signaling promotes lipid synthesis and enhances NADPH production, which buffers oxidative stress and provides resistance against lipid peroxidation [29]. However, inhibition of this pathway reduces NADPH availability, thereby weakening ferroptosis defenses. Similarly, alterations in the SWI/SNF complex, particularly ARID1A and SMARCB1, have been associated with ferroptosis sensitivity in other malignancies by impairing redox homeostasis and glutathione metabolism [30]. These findings suggest that chromatin remodeling defects in ATC may also confer vulnerability to ferroptosis, although further direct studies are warranted.

### 2.2. Iron Metabolism Dysregulation

Ferroptosis is fundamentally an iron-driven process, and dysregulation of iron metabolism is a hallmark of ATC. ATC cells frequently exhibit elevated expression of the transferrin receptor (TFRC/CD71), which enhances iron uptake while simultaneously increasing intracellular labile iron pools [31,32]. At the same time, iron storage proteins such as ferritin heavy chain (FTH1) and ferritin light chain (FTL) act as buffers, sequestering excess iron and protecting against ferroptosis [33].

A critical mechanism linking iron metabolism to ferroptosis is ferritinophagy, a selective form of autophagy mediated by nuclear receptor coactivator 4 (NCOA4), which delivers ferritin to the lysosome for degradation [34,35]. This process releases stored iron, thereby increasing the labile iron pool and sensitizing cells to ferroptosis. In ATC, ferritinophagy has been implicated in the mechanism by which vitamin C induces ferroptotic death. By destabilizing ferritin and increasing free Fe^2+^, vitamin C amplifies Fenton chemistry and lipid peroxidation, leading to ferroptosis [13]. Genetic regulators such as SIRT6 also intersect with ferritinophagy; SIRT6 upregulation enhances NCOA4-mediated ferritin degradation and potentiates ferroptosis in ATC xenografts [36].

Another layer of regulation comes from heme metabolism. ATC cells frequently overexpress heme oxygenase-1 (HO-1), which degrades heme into biliverdin, carbon monoxide, and free iron (Fe^2+^) [37]. The role of HO-1 in ferroptosis is paradoxical: under certain conditions, HO-1 upregulation increases intracellular iron and promotes ferroptosis, while in other contexts, it provides cytoprotection through antioxidant effects [38]. In thyroid cancer models treated with natural compounds such as curcumin, HO-1 induction appeared to contribute to ferroptotic death, while its inhibition enhanced sensitivity to ferroptosis, underscoring the dual nature of this enzyme in redox biology [39].

Together, these findings demonstrate that iron metabolism in ATC is dynamically regulated by both iron acquisition and storage pathways, and that ferritinophagy represents a pivotal mechanism tipping the balance toward ferroptotic vulnerability.

### 2.3. Lipid Metabolism Remodeling

The execution of ferroptosis requires peroxidation of polyunsaturated fatty acids (PUFAs) within membrane phospholipids. Enzymes such as acyl-CoA synthetase long-chain family member 4 (ACSL4) and lysophosphatidylcholine acyltransferase 3 (LPCAT3) mediate the incorporation of arachidonic acid and adrenic acid into phosphatidylethanolamines (PEs), creating lipid species highly susceptible to peroxidation [40,41,42]. In ATC, upregulation of ACSL4 has been observed, suggesting that the tumor membrane landscape is enriched with PUFA-containing phospholipids, thereby predisposing cells to ferroptosis [43]. Conversely, ACSL3-mediated incorporation of monounsaturated fatty acids (MUFAs) confers resistance by displacing PUFAs from membranes, highlighting the opposing roles of ACSL4 and ACSL3 in determining ferroptosis susceptibility [44,45].

Recent studies have also highlighted the contribution of peroxisomal metabolism and plasmalogen synthesis in ferroptosis regulation. Plasmalogens are ether-linked phospholipids that contain PUFA chains and are particularly enriched in cellular membranes vulnerable to peroxidation [46]. Peroxisomes are required for plasmalogen biosynthesis, and ATC cells with high peroxisomal activity may therefore be more prone to ferroptosis [47]. Conversely, metabolic rewiring that favors MUFA production or cholesterol biosynthesis can provide resistance, as these lipid species are less susceptible to peroxidation [48].

The dynamic remodeling of lipid metabolism in ATC, particularly during epithelial-to-mesenchymal transition (EMT), further enhances ferroptosis sensitivity [49]. EMT-associated transcription factors reprogram lipid metabolic pathways, leading to increased PUFA incorporation and higher ROS dependency, creating an environment in which ferroptosis induction becomes particularly effective.

### 2.4. Antioxidant Defense Systems in ATC

To counteract the threat of lipid peroxidation, ATC cells deploy multiple antioxidant defense systems. The GPX4–GSH axis remains the most prominent. GPX4 detoxifies lipid peroxides using GSH as a cofactor, and its expression is upregulated in thyroid cancer tissues, including ATC, where it correlates with aggressive progression [50]. Pharmacological inhibition of GPX4 invariably results in ferroptotic death, confirming its central role as a ferroptosis checkpoint [51]. The system Xc^−^ transporter (SLC7A11/SLC3A2) replenishes intracellular cysteine for GSH synthesis. ATC cells with high SLC7A11 expression display greater resistance to oxidative stress and ferroptosis [52]. Experimental inhibition of system Xc^−^ by erastin or sulfasalazine reduces GSH levels and sensitizes ATC cells to ferroptotic death, suggesting that cystine import is a key vulnerability [53].

Additional layers of defense include the FSP1–CoQ_10_ axis, which regenerates ubiquinol as a lipid radical-trapping antioxidant, and mitochondrial DHODH, which prevents lipid peroxidation in the inner mitochondrial membrane [54,55,56]. While these pathways have not been directly studied in ATC, their relevance is inferred from other cancers, and preliminary data suggest that ATC’s high mitochondrial activity could make DHODH particularly important. The GCH1–BH_4_ axis also contributes to ferroptosis resistance by generating tetrahydrobiopterin (BH_4_), which functions as a potent antioxidant [57]. Finally, the thioredoxin (TXN) system, though less studied in ATC, provides an additional antioxidant layer by maintaining redox balance independently of GSH [58,59]. Its activity may compensate when GSH levels are depleted, and targeting this system could further sensitize ATC cells to ferroptosis [60].

### 2.5. Nrf2 Signaling and Redox Adaptation

The Nrf2–KEAP1 pathway orchestrates a broad antioxidant response and represents a critical regulator of ferroptosis resistance [61,62]. Nrf2 transcriptionally upregulates GPX4, SLC7A11, FTH1, FTL, and HO-1, thereby reinforcing defenses against lipid peroxidation [63,64]. In ATC, aberrant Nrf2 activation has been observed, often driven by oncogenic stress or KEAP1 loss-of-function alterations [65,66]. Importantly, pharmacological studies in thyroid cancer cell models show that suppression of Nrf2 or its downstream effector HO-1 enhances sensitivity to ferroptosis inducers such as curcumin and neferine [14,39].

The role of Nrf2 is also closely intertwined with EMT [67]. ATC progression involves dedifferentiation and acquisition of mesenchymal traits, which elevate ROS levels and increase dependency on antioxidant defenses [68,69]. In this setting, Nrf2 activation serves as a survival mechanism but also creates a therapeutic vulnerability: inhibiting Nrf2 during EMT may push ATC cells beyond their oxidative threshold and trigger ferroptosis [66,70]. This duality underscores the importance of considering tumor stage and differentiation status when evaluating ferroptosis-based strategies in ATC.

### 2.6. EMT, Metabolic Rewiring, and Ferroptosis Sensitivity

EMT is a hallmark of ATC progression, enabling invasion, metastasis, and therapeutic resistance. EMT is accompanied by profound metabolic rewiring, including increased mitochondrial respiration, elevated ROS production, and reprogrammed lipid metabolism [71,72]. These changes enhance the reliance of ATC cells on antioxidant systems such as GPX4 and Nrf2, thereby creating a state of conditional vulnerability to ferroptosis [73]. Preclinical studies in other cancers have demonstrated that EMT-associated transcriptional programs sensitize cells to ferroptosis by increasing PUFA incorporation into membranes and elevating oxidative stress [73,74,75]. Similar mechanisms are likely operative in ATC, where dedifferentiation and EMT correlate with heightened sensitivity to ferroptosis inducers [10]. Recent evidence further highlights the role of receptor tyrosine kinases in coordinating metabolic rewiring and ferroptosis in ATC. RON receptor tyrosine kinase was found to be highly expressed in thyroid cancer cells, where its inhibition suppressed glycolysis, promoted ferroptosis, and enhanced chemosensitivity [43]. Mechanistically, RON interference disrupted MAPK/CREB signaling, leading to reduced glucose uptake, lactate production, and expression of glycolytic enzymes such as GLUT1, HK2, and PKM2, thereby lowering antioxidant capacity and increasing ferroptotic vulnerability.

In summary, ATC is characterized by a molecular environment that both predisposes to and defends against ferroptosis. Genomic alterations such as TP53, RAS, BRAF^V600E^, PIK3CA, and ARID1A influence ferroptosis sensitivity through redox and metabolic pathways. Dysregulation of iron metabolism, particularly via ferritinophagy and HO-1 activity, increases labile iron pools that can fuel ferroptosis. Lipid metabolism remodeling through ACSL4, LPCAT3, and peroxisome-derived plasmalogens enriches membranes with oxidizable PUFAs. Antioxidant defense systems, including GPX4, system Xc^−^, FSP1, DHODH, GCH1–BH_4_, and the thioredoxin system, counteract ferroptosis, while Nrf2 activation and EMT further modulate vulnerability. Collectively, these mechanisms position ATC as a malignancy in which ferroptosis is both a natural liability and a tightly regulated threat, providing a strong rationale for therapeutic exploitation.

## 3. Preclinical and Experimental Evidence of Ferroptosis in ATC

### 3.1. Pharmacological Inducers of Ferroptosis

Pharmacological agents represent some of the earliest tools used to probe ferroptosis in ATC and have yielded critical insights into the feasibility of this approach (Table 1). Among them, vitamin C has been especially well studied. In the ATC cell line 8505C, high-dose vitamin C was shown to activate ferritinophagy, thereby degrading ferritin and liberating iron into the labile pool [13]. This influx of Fe^2+^ amplified the Fenton reaction, producing hydroxyl radicals and increasing lipid peroxidation. Importantly, ferrostatin-1 rescued vitamin C-induced cell death, establishing ferroptosis as the underlying mechanism rather than other forms of oxidative damage. Beyond short-term cytotoxicity, vitamin C also impaired clonogenic survival, suggesting that ferroptosis induction can provide durable suppression of ATC cell growth.

Natural products have also been implicated as ferroptosis inducers. Tenacissoside H (TDH), derived from *Marsdenia tenacissima*, suppressed the proliferation, invasion, and survival of ATC cells *in vitro* [76]. Mechanistically, TDH reduced the expression of GPX4, SLC7A11, HO-1, and transferrin receptor while increasing lipid ROS accumulation. The addition of ferrostatin-1 reversed these effects, confirming that ferroptosis was the dominant mode of death. The in vivo xenograft models treated with TDH demonstrated significantly decreased tumor volume and reduced metastatic potential. Shikonin, a naphthoquinone derivative derived from traditional Chinese medicine, was also shown to inhibit ATC cell growth by simultaneously promoting ferroptosis and suppressing glycolysis [60]. In CAL-62 and 8505C cells, shikonin reduced the expression of GPX4, thioredoxin reductase 1 (TXNRD1), and glycolytic regulators such as PKM2 and GLUT1, thereby elevating ROS and impairing glucose metabolism. The *in vivo* xenograft studies further confirmed tumor growth inhibition, highlighting shikonin as a dual-action agent targeting both redox and metabolic vulnerabilities in ATC. Other natural compounds, including neferine and curcumin, were found to sensitize thyroid cancer cells to ferroptosis through suppression of the Nrf2/HO-1/NQO1 axis, reduction in GPX4 activity, and increased oxidative stress [14,39]. These findings support the hypothesis that natural agents with dual antioxidant–prooxidant roles can destabilize the redox homeostasis of ATC cells, pushing them beyond the threshold of ferroptosis.

### 3.2. Targeted Therapies Combined with Ferroptosis Inducers

The therapeutic relevance of ferroptosis in ATC becomes most evident when considered in the context of targeted therapies. BRAF^V600E^ mutations, common in ATC, are clinically actionable with BRAF inhibitors such as dabrafenib [81,82]. However, the development of resistance remains nearly universal. Preclinical models have shown that BRAF inhibitor-resistant ATC cells display heightened sensitivity to ferroptosis inducers, including RSL3, ML162, and imidazole ketone erastin (IKE) [77]. Combination therapy led to significant increases in lipid ROS, higher intracellular iron, and suppression of ferroportin-1 expression, thereby driving lethal iron accumulation. In orthotopic ATC models, dabrafenib combined with GPX4 inhibitors produced more profound tumor regression than either therapy alone, highlighting ferroptosis as a strategy to overcome resistance [77].

Anlotinib, a multitargeted tyrosine kinase inhibitor, also exerts direct ferroptosis-inducing activity in ATC [12,78]. Treatment of 8505C, KHM-5M, and C643 cells with anlotinib increased ROS production, reduced GPX4 and ferritin levels, and activated ER stress via PERK and CHOP [78]. Interestingly, anlotinib simultaneously activated autophagy, which acted as a cytoprotective mechanism. When autophagy was pharmacologically blocked, anlotinib-induced ferroptosis intensified, leading to superior tumor control in xenografts compared with anlotinib alone [12]. These findings suggest that targeting ferroptosis pathways can enhance the efficacy of existing targeted therapies while also addressing resistance mechanisms.

### 3.3. Genetic Regulators of Ferroptosis in ATC

ATC studies have uncovered several intrinsic regulators that modulate ferroptosis sensitivity. SIRT6, a histone deacetylase involved in metabolic regulation, has been identified as a potent sensitizer of ferroptosis [36]. Overexpression of SIRT6 promoted NCOA4-dependent ferritinophagy, thereby elevating labile iron and enhancing ferroptotic susceptibility. Knockout of SIRT6 conferred resistance to inducers such as RSL3 and erastin, while xenografts with elevated SIRT6 expression responded strongly to sulfasalazine, a system Xc^−^ inhibitor.

The GPR34–USP8 axis also modulates ferroptosis in ATC [79]. GPR34 was found to be aberrantly overexpressed, and its stabilization by the deubiquitinase USP8 suppressed ferroptosis, enhancing tumor survival. Inhibition of USP8 with DUB-IN-3 destabilized GPR34, restored ferroptosis, and suppressed tumor growth in xenograft models. These findings identify novel molecular circuits that govern ferroptosis in ATC and may represent therapeutic targets.

Furthermore, hyperactivation of Nrf2 is a well-established resistance mechanism. Nrf2 upregulates GPX4, SLC7A11, and HO-1, bolstering antioxidant defenses. Pharmacological inhibition of Nrf2 or HO-1 sensitized ATC cells to ferroptosis induced by natural products such as neferine and curcumin, highlighting Nrf2 as a critical determinant of ferroptosis resistance [14,66,70].

In addition, recent work identified eukaryotic translation initiation factor 3 subunit H (EIF3H) as a novel regulator of ferroptosis resistance in ATC [80]. EIF3H acts as a deubiquitinating enzyme that interacts with and stabilizes β-catenin, thereby sustaining Wnt/β-catenin signaling. Knockdown of EIF3H disrupted this pathway, leading to reduced proliferation, invasion, and ferroptosis resistance in ATC cells. Mechanistically, EIF3H dysregulation was further linked to m^6^A modification and the m^6^A reader IGF2BP2, suggesting an epitranscriptomic layer of ferroptosis control. These findings implicate the EIF3H/β-catenin axis as both a diagnostic marker and therapeutic target in ATC.

### 3.4. Nanotechnology-Driven Ferroptosis Strategies

Nanoplatforms have emerged as innovative vehicles for inducing ferroptosis in ATC. A notable example is the Fe^3+^Cur-PFP@IR780-LIP (FCIPL) system, which encapsulates iron and curcumin within a liposomal structure responsive to ultrasound cavitation [15]. Upon ultrasound stimulation, FCIPL penetrated deeply into ATC tumors, released Fe^2+^ and curcumin directly into mitochondria, and initiated a cascade of lipid peroxidation described as “domino-ferroptosis.” This was coupled with sonodynamic therapy, amplifying tumor cell killing. In vivo, the nanoplatform suppressed tumor growth and enabled multimodal imaging, including MRI, photoacoustic, ultrasound, and fluorescence modalities. Such technologies represent a step toward integrating ferroptosis induction with precision drug delivery and real-time monitoring.

### 3.5. Mechanisms of Resistance to Ferroptosis-Targeted Therapy in ATC

Ferroptosis-targeted therapies in ATC encounter several ATC-specific resistance mechanisms that reduce treatment efficacy. In particular, persistent activation of Nrf2, upregulation of system Xc^−^ (SLC7A11), and GPX4 overexpression create a robust antioxidant environment that suppresses lipid peroxide accumulation. Metabolic rewiring—including enhanced glutathione synthesis, NADPH regeneration, and incorporation of monounsaturated fatty acids via ACSL3—further reinforces intrinsic ferroptosis resistance in ATC. These mechanisms underscore the need for combination strategies capable of overcoming these redox and metabolic barriers.

Despite robust preclinical evidence, ATC cells display heterogeneity in ferroptosis responses. Comparative studies revealed that 8505C ATC cells tolerated iron overload-induced ferroptosis better than follicular thyroid carcinoma cells, a phenotype linked to upregulation of transferrin receptor CD71 and adaptive iron handling [53]. This underscores the challenge of intratumoral heterogeneity, where subsets of cells may develop compensatory mechanisms to evade ferroptosis. Understanding these mechanisms is essential for developing combination therapies that can suppress resistance and achieve more durable responses.

In summary, preclinical studies demonstrate that ferroptosis can be pharmacologically, genetically, and technologically induced in ATC, with promising anti-tumor effects. However, heterogeneity and adaptive resistance highlight the need for biomarker-driven strategies and rational combinations to maximize therapeutic efficacy.

## 4. Therapeutic Strategies for Exploiting Ferroptosis in ATC

### 4.1. Initiators of Ferroptosis

**Figure 3:**
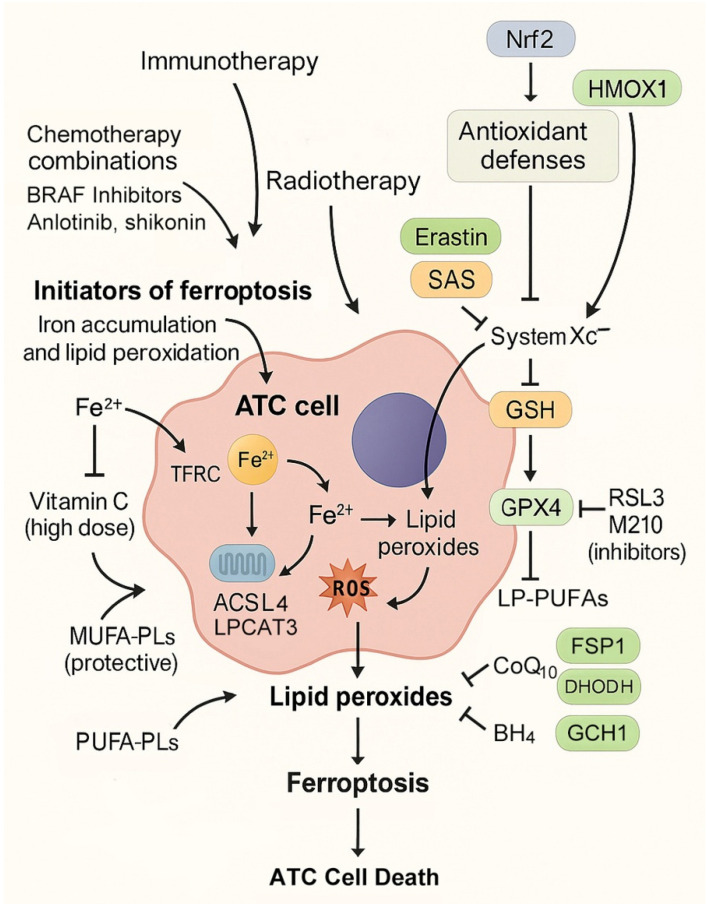
Therapeutic strategies for exploiting ferroptosis in anaplastic thyroid cancer (ATC). Ferroptosis in ATC is initiated by iron accumulation and lipid peroxidation. Transferrin receptor (TFRC)-mediated Fe^3+^ uptake, NCOA4-dependent ferritinophagy, and high-dose vitamin C expand the labile Fe^2+^ pool, fueling ROS generation via Fenton reactions. ACSL4 and LPCAT3 drive incorporation of PUFAs into phosphatidylethanolamines (PEs), sensitizing membranes to lipid peroxidation, while ACSL3 promotes MUFA-PLs that are protective. Ferroptosis occurs when ROS-driven lipid peroxides accumulate beyond the capacity of detoxification systems. Antioxidant defenses include the System Xc^−^–GSH–GPX4 axis, inhibited by erastin, sulfasalazine (SAS), RSL3, and ML210. Parallel protective pathways involve FSP1–CoQ_10_H_2_, DHODH–CoQ_10_H_2_, and GCH1–BH_4_. Nrf2 enhances resistance by upregulating GPX4, SLC7A11, HO-1, and ferritin. Combination regimens incorporating BRAF inhibitors, chemotherapy (e.g., isobavachalcone with doxorubicin), radiotherapy, immunotherapy (via IFN-γ-mediated SLC7A11 suppression), and anlotinib with autophagy inhibitors offer synergistic opportunities to overcome therapy resistance in ATC.

Therapeutic induction of ferroptosis in ATC can be achieved by amplifying oxidative stress through iron accumulation and lipid peroxidation (Table 2, Figure 3). One mechanism is pharmacological stimulation of ferritinophagy, which mobilizes stored ferritin-bound iron into the labile pool, thereby fueling Fenton chemistry [83]. High-dose vitamin C exemplifies this strategy in ATC models, where ferritin degradation markedly increased lipid ROS and ferroptotic cell death [13]. Agents that directly elevate iron uptake via transferrin receptor upregulation or disrupt ferritin stability are also promising candidates.

At the level of lipid metabolism, ferroptosis induction requires phospholipid substrates enriched with PUFAs. Upregulation of ACSL4 and LPCAT3 promotes incorporation of arachidonic and adrenic acid into phosphatidylethanolamines, making membranes highly peroxidizable [84,85]. Experimental studies in thyroid cancer models have demonstrated that ACSL4 expression correlates with ferroptosis sensitivity, whereas resistance is associated with ACSL3-driven MUFA enrichment [41,45]. In ATC, which undergoes profound metabolic rewiring and EMT, PUFA enrichment of membranes appears particularly pronounced, creating a metabolic landscape conducive to ferroptosis induction [86].

Therapeutic strategies aimed at increasing peroxisome-derived plasmalogens may further sensitize ATC to ferroptosis, given their susceptibility to oxidation [87,88]. Conversely, metabolic interventions that block MUFA synthesis or cholesterol biosynthesis could potentiate ferroptosis by eliminating competing, less oxidizable substrates. Thus, pharmacological initiators, dietary interventions, and metabolic modulators converge on the principle of overwhelming ATC antioxidant capacity by expanding the pool of oxidizable lipids in the presence of abundant iron.

### 4.2. Blockers of Antioxidant Defenses

If initiators “ignite the spark” of ferroptosis, then blocking antioxidant defenses ensures that the fire cannot be extinguished. The most well-established strategy is targeting GPX4, the selenoenzyme responsible for detoxifying lipid peroxides. Direct GPX4 inhibitors such as RSL3 and ML210 have induced ferroptosis in ATC models, while indirect inhibition can be achieved by blocking system Xc^−^, thereby depleting cysteine and impairing GSH synthesis. The system Xc^−^ inhibitor sulfasalazine, for instance, suppressed ATC xenograft growth in the presence of SIRT6 upregulation [36].

Additional layers of defense provide redundancy. The FSP1–CoQ_10_ axis regenerates reduced ubiquinol, functioning as a lipid radical scavenger. Inhibition of FSP1 has been shown in other cancers to synergize with GPX4 blockade, suggesting potential for ATC [89]. The mitochondrial enzyme DHODH similarly regenerates ubiquinol within the inner mitochondrial membrane, and dual inhibition of DHODH and GPX4 has been reported to induce profound ferroptosis in other tumor types [56,90]. The GCH1–BH_4_ pathway, which generates tetrahydrobiopterin, represents yet another defense, with BH_4_ serving as a potent radical-trapping antioxidant [57]. Although these mechanisms have not yet been studied directly in ATC, their established roles in other malignancies and the high mitochondrial activity of ATC suggest strong translational relevance.

Finally, targeting Nrf2 offers a unifying strategy. Aberrant Nrf2 activation is frequent in ATC and confers ferroptosis resistance by upregulating GPX4, SLC7A11, HO-1, and ferritin subunits [62,91,92]. Pharmacological inhibition of Nrf2 or its effectors has been shown to sensitize ATC cells to ferroptosis inducers such as neferine and curcumin [14,39,66]. Thus, disabling antioxidant defenses, particularly in the context of Nrf2-driven adaptation, remains central to ferroptosis-based therapy in ATC.

### 4.3. Combination Regimens

Given ATC’s notorious resistance to monotherapy, ferroptosis-targeted strategies will likely achieve their greatest success when integrated into combination regimens [93]. One major avenue involves targeted therapy combinations. BRAF^V600E^-mutant ATC responds transiently to dabrafenib and trametinib, but relapse occurs due to acquired resistance [94,95]. Preclinical evidence demonstrates that ferroptosis inducers resensitize resistant cells, producing tumor regression when combined with BRAF inhibitors [77]. Similarly, anlotinib, which inherently induces ferroptosis, shows superior efficacy when paired with autophagy inhibitors, as suppression of protective autophagy unmasks its full ferroptotic potential [12].

Radiotherapy, a mainstay of ATC management, generates ROS and induces lipid peroxidation [3,96]. While ATC is often relatively radioresistant, GPX4 inhibition or cystine deprivation may amplify radiation-induced oxidative stress, driving ferroptotic death [25]. Preclinical validation of this combination remains limited, but the biological rationale is compelling. Immunotherapy also intersects with ferroptosis [97,98]. IFN-γ released from CD8^+^ T cells downregulates SLC7A11, sensitizing tumor cells to ferroptosis [99]. This finding suggests that combining immune checkpoint blockade with ferroptosis induction could produce synergistic anti-tumor effects. In the context of ATC, which often displays an inflamed tumor microenvironment, this strategy holds translational promise [100].

In addition to targeted therapy, radiotherapy, and immunotherapy, chemotherapy-based combinations have also been explored. The natural chalcone derivative isobavachalcone (IBC) synergized with doxorubicin to suppress ATC progression by activating ferroptosis [52]. In CAL-62 and 8505C cells, the combination increased iron, ROS, and malondialdehyde (MDA) while depleting GSH and reducing GPX4 and SLC7A11 expression. Ferrostatin-1 rescued these effects, confirming ferroptosis as the mechanism. In vivo xenograft models also demonstrated enhanced tumor suppression, highlighting IBC plus doxorubicin as a potential ferroptosis-based chemotherapy regimen for ATC.

Together, these combinations demonstrate that ferroptosis is not an isolated pathway but a mechanism that can be strategically integrated with existing therapies to overcome resistance and enhance efficacy.

### 4.4. Drug Repurposing and Natural Compounds

Drug repurposing provides a pragmatic route toward clinical translation. Sulfasalazine, approved for rheumatoid arthritis and inflammatory bowel disease, has demonstrated preclinical efficacy against ATC xenografts when combined with SIRT6 overexpression [36]. Statins, widely prescribed for hyperlipidemia, inhibit the mevalonate pathway, thereby reducing CoQ_10_ levels and impairing antioxidant defenses [101]. Artesunate, an antimalarial agent, generates iron-dependent ROS and has been shown to induce ferroptosis in multiple tumor types; its application to ATC warrants exploration [102].

Natural compounds remain an attractive adjunct, as many exhibit dual roles in modulating oxidative stress. Curcumin, neferine, and TDH have all been demonstrated to reduce GPX4 or SLC7A11 activity, increase ROS, and suppress ATC tumor growth in vivo [14,39,76]. These agents may be particularly useful in combination with targeted therapy, immunotherapy, or radiotherapy, where their ferroptosis-promoting effects could be synergistically amplified.

### 4.5. Challenges in Clinical Translation

Despite these promising strategies, multiple challenges remain before ferroptosis-based therapy can enter clinical practice for ATC [103]. The most immediate concern is systemic toxicity [18]. Ferroptosis relies on iron-driven lipid peroxidation, which can damage normal tissues rich in PUFAs, including the brain, heart, and kidneys. Careful dose titration, biomarker-guided patient selection, and targeted delivery systems are therefore essential.

Biomarker development is critical to stratify patients who are most likely to benefit. Potential candidates include GPX4, SLC7A11, ACSL4, and ferritinophagy markers such as NCOA4 [73]. Multi-omics approaches integrating genomics, lipidomics, and proteomics will be required to establish reliable predictive panels. Drug delivery represents another obstacle. Many ferroptosis inducers have limited tumor specificity [104,105]. Nanotechnology platforms, such as FCIPL, demonstrate how ferroptosis inducers can be selectively delivered to ATC tumors while simultaneously enabling real-time imaging [15]. However, these technologies remain at the preclinical stage and require rigorous validation. Finally, resistance mechanisms threaten to undermine long-term efficacy. Adaptive responses such as Nrf2 hyperactivation, lipid remodeling toward MUFAs, and compensatory upregulation of ferritin or CD71 allow ATC cells to withstand ferroptotic stress [47,53,65]. Overcoming these adaptations will require rationally designed combination regimens, ideally integrating ferroptosis inducers with inhibitors of resistance pathways.

In conclusion, therapeutic exploitation of ferroptosis in ATC encompasses a spectrum of strategies, from initiating lipid peroxidation and iron overload to disabling redundant antioxidant defenses. The most effective approaches are likely to involve rational combinations with targeted therapy, radiotherapy, and immunotherapy, supported by biomarker-driven patient selection and innovative delivery systems. While challenges remain in toxicity management and resistance, the convergence of molecular insights, preclinical validation, and technological innovation positions ferroptosis as one of the most promising avenues for addressing the therapeutic impasse of ATC.

## 5. Biomarkers, Prognostic Indicators, and Patient Selection for Ferroptosis-Based Therapy in ATC

### 5.1. Genetic Markers and Ferroptosis-Related Genes

The identification of genetic biomarkers that predict ferroptosis sensitivity is critical for developing precision medicine strategies in ATC. Large-scale genomic studies have shown that ATC carries frequent mutations in TP53, TERT promoter, BRAF, RAS, and PI3K/AKT pathway regulators, all of which intersect with ferroptosis pathways in diverse ways [1,8]. For example, loss of TP53 function alters cystine uptake and redox control by deregulating the expression of SLC7A11, a central component of system Xc^−^, thereby shifting the balance toward ferroptosis susceptibility [21,106]. Similarly, RAS mutations, detected in up to 40% of poorly differentiated thyroid cancers and approximately 20% of ATCs, are known to enhance oxidative stress and lipid metabolism, rendering tumor cells dependent on antioxidant defenses [107]. These genetic contexts are strongly associated with vulnerability to ferroptosis-inducing compounds such as erastin and GPX4 inhibitors [10].

Beyond canonical driver mutations, transcriptional regulators and chromatin modifiers also serve as biomarkers of ferroptosis response. For instance, SIRT6 has been identified as a sensitizer of ferroptosis through its ability to promote NCOA4-dependent ferritinophagy, thereby elevating intracellular iron levels [36]. Overexpression of SIRT6 correlated with increased sensitivity to sulfasalazine and GPX4 inhibitors, whereas loss of SIRT6 reduced susceptibility [36], suggesting that SIRT6 expression levels may function as a predictive biomarker for ferroptosis-based therapy in ATC.

### 5.2. Protein and Enzymatic Regulators as Biomarkers

Several protein markers have emerged as direct indicators of ferroptosis activity in ATC. GPX4, the master lipid peroxide detoxifying enzyme, is consistently upregulated in thyroid cancers, including ATC, and its overexpression correlates with poor prognosis [108]. Inhibition or genetic silencing of GPX4 reliably induces ferroptosis in ATC cells, establishing GPX4 expression as a biomarker of both prognosis and therapeutic responsiveness [13,52]. Similarly, high expression of SLC7A11 predicts aggressive behavior and resistance to multiple therapies [109]. Inhibition of this transporter with erastin or sulfasalazine has been shown to resensitize ATC cells to ferroptosis [28,53].

Ferritin subunits, particularly FTH1 and FTL, also serve as important biomarkers. Elevated ferritin levels buffer intracellular iron and correlate with resistance to ferroptosis. Conversely, activation of ferritinophagy by NCOA4 increases the labile iron pool and enhances ferroptotic death, positioning ferritin dynamics as both a prognostic factor and a therapeutic target [36]. Other proteins, such as HO-1 and transferrin receptor 1 (CD71), may also function as biomarkers; HO-1 upregulation has a dual role, at times protective but under certain conditions pro-ferroptotic, while CD71 has been implicated in iron-handling adaptations that modulate ferroptosis sensitivity [37,53].

### 5.3. Lipidomic and Metabolic Signatures

The susceptibility of ATC cells to ferroptosis is fundamentally linked to lipid composition. ACSL4 and LPCAT3 drive the incorporation of polyunsaturated fatty acids (PUFAs) into phospholipids, priming membranes for lipid peroxidation [84]. High expression of ACSL4 has been correlated with ferroptosis sensitivity, while increased ACSL3-mediated monounsaturated fatty acid incorporation confers resistance [41,44]. Lipidomic profiling of ATC cells could therefore provide predictive biomarkers of ferroptosis response.

Metabolic markers, such as levels of GSH, NADPH, and CoQ_10_ (ubiquinol), also serve as key determinants. For instance, tumors with depleted GSH pools or impaired NADPH regeneration are more prone to ferroptosis, whereas elevated mevalonate pathway activity and ubiquinol levels confer protection [7]. Such metabolic signatures can be measured by transcriptomic or metabolomic assays and may guide patient stratification for ferroptosis-based interventions.

### 5.4. Immune Microenvironmental Correlations

The tumor immune microenvironment has an increasingly recognized role in ferroptosis regulation. In ATC, which often displays immune infiltration, interferon-γ secreted by CD8^+^ T cells has been shown to downregulate SLC7A11, thereby promoting ferroptosis in tumor cells [99]. Conversely, tumor-associated macrophages (TAMs), particularly the M2 phenotype, may release cytokines that buffer oxidative stress and reduce ferroptosis susceptibility [110]. Additionally, ferroptotic cells release danger-associated molecular patterns (DAMPs), which can modulate immune responses and potentially synergize with immunotherapies [111]. Thus, immune signatures, such as the density of CD8^+^ T cells, the polarization of macrophages, or the expression of interferon-stimulated genes, may serve as indirect biomarkers of ferroptosis responsiveness in ATC.

### 5.5. Translational Challenges and Opportunities

Although several biomarkers have been proposed, their clinical implementation remains challenging. Most studies have been conducted in cell lines or xenograft models, and validation in patient-derived ATC samples is limited. The rarity of ATC further complicates large-scale biomarker discovery. Another challenge lies in the dual roles of certain molecules; for example, HO-1 may act as both a pro- and anti-ferroptotic factor depending on context, making its interpretation as a biomarker complex [37]. Similarly, discrepancies in transferrin receptor expression across thyroid cancer subtypes highlight the heterogeneity of ferroptosis regulation [32].

Nonetheless, the integration of multi-omics technologies, including genomics, transcriptomics, proteomics, and lipidomics, holds promise for developing composite biomarker panels. Such panels could stratify patients into ferroptosis-sensitive and -resistant groups, thereby guiding therapeutic decisions. For instance, patients with high GPX4 and SLC7A11 expression combined with low ACSL3 and high ACSL4 signatures may represent ideal candidates for ferroptosis-inducing therapies, consistent with preclinical evidence showing that GPX4 and SLC7A11 overexpression confers ferroptosis resistance [50,73], whereas ACSL3-mediated MUFA enrichment promotes resistance [45] and ACSL4 upregulation increases ferroptosis sensitivity [41,43]. Future clinical trials should incorporate biomarker-driven patient selection to maximize efficacy and minimize toxicity in the treatment of ATC.

## 6. Conclusions and Perspectives

ATC remains among the most lethal human cancers, with limited survival despite surgery, chemotherapy, radiotherapy, and even targeted and immune-based treatments [112]. This reality underscores the urgent need for novel therapeutic paradigms. Ferroptosis, an iron-dependent form of regulated cell death characterized by lipid peroxidation and oxidative damage, has emerged as a promising vulnerability in ATC.

Preclinical studies demonstrate that ferroptosis can be induced in ATC through diverse strategies, including small molecules, natural compounds, targeted therapies, and genetic regulators. These interventions not only suppress proliferation in vitro but also inhibit tumor growth in vivo, supporting translational feasibility. Importantly, recent findings highlight the interplay between ferroptosis, metabolic rewiring, oncogenic signaling, and drug resistance, suggesting that ferroptosis-targeted approaches could be integrated into combination regimens to resensitize ATC to conventional and targeted treatments.

Despite this promise, key challenges remain. Systemic toxicity poses a major concern, particularly in lipid-rich tissues such as the nervous system and myocardium. Strategies such as optimized dosing, targeted delivery, and nanoplatforms may help widen the therapeutic window, but clinical validation is still lacking. Another obstacle is the heterogeneity of ferroptosis responses in ATC, underscoring the importance of biomarker-driven patient selection. Multi-omics approaches will be essential to identify predictive signatures and refine therapeutic stratification, while the immune contexture should be incorporated to guide rational combinations with immunotherapy. Looking ahead, three priorities stand out: rigorous preclinical validation in patient-derived models, biomarker discovery embedded in translational studies, and early-phase clinical trials of ferroptosis-targeted agents and rational combinations. Achieving these goals will require cross-disciplinary collaboration spanning oncology, molecular biology, pharmacology, and bioengineering.

In addition, future research should explore rational combination strategies that integrate ferroptosis induction with existing therapeutic modalities. Preclinical evidence already supports the synergy between ferroptosis activation and radiotherapy [96], immune checkpoint inhibition via IFN-γ-mediated SLC7A11 suppression [99], and chemotherapy regimens such as isobavachalcone plus doxorubicin [52]. Moreover, metabolic co-targeting—such as combining GPX4 inhibition with glutaminolysis blockers or mevalonate pathway suppression—may overcome intrinsic resistance to ferroptosis-based therapy. Nanoparticle-mediated delivery systems also represent a promising direction, enhancing tumor specificity while reducing systemic toxicity [104,105]. Together, these advances highlight the therapeutic potential of ferroptosis-centered combination regimens and underscore the importance of continued translational research to bring ferroptosis-targeted therapies into clinical practice for ATC.

In conclusion, ferroptosis offers a mechanistically distinct and therapeutically exploitable vulnerability in ATC. By integrating ferroptosis induction with existing treatment modalities and leveraging biomarker-guided strategies, it may be possible to overcome resistance and improve outcomes in this otherwise intractable cancer.

## Figures and Tables

**Table 1:** Preclinical studies of ferroptosis in anaplastic thyroid cancer (ATC).

Authors (Year)	Model	Intervention	Mechanism	Key Findings	References
Wang et al. (2021)	8505C cells	Vitamin C	Ferritinophagy, Fe^2+^ release, lipid ROS	Ferroptotic death, rescued by ferrostatin-1	[13]
He et al. (2025)	ATC cells, xenograft	Tenacissoside H	↓ GPX4, ↓ SLC7A11, ↑ ROS	Reduced proliferation, invasion, tumor growth	[76]
Li et al. (2023)	ATC cells	Neferine	Inhibition of Nrf2/HO-1/NQO1	Enhanced lipid peroxidation, ferroptosis	[14]
Chen et al. (2024)	ATC cells	Curcumin	HO-1 activation, ↓ GPX4	Ferroptotic sensitivity, reduced growth	[39]
Noronha et al. (2025)	ATC cells, orthotopic model	BRAF inhibitor + GPX4 inhibitor	↑ Lipid ROS, ↓ FPN1	Overcome dabrafenib resistance, tumor regression	[77]
Guo et al. (2025); Wu et al. (2023)	ATC cells, xenograft	Anlotinib	↑ ROS, ↓ GPX4, PERK–CHOP ER stress	Ferroptosis induction, amplified by autophagy inhibition	[12,78]
Yang et al. (2023)	ATC cells, xenograft	SIRT6 + sulfasalazine	↑ Ferritinophagy (NCOA4), ↓ system Xc^−^	Sensitized to ferroptosis, tumor suppression	[36]
Yan et al. (2025)	ATC cells, xenograft	USP8 inhibitor (DUB-IN-3)	↓ GPR34 stabilization	Restored ferroptosis, suppressed tumor growth	[79]
Dong et al. (2025)	ATC xenograft	FCIPL nanoplatform	Fe^2+^ release + curcumin, mitochondrial lipid ROS	Domino-ferroptosis, sonodynamic synergy	[15]
Yang et al. (2024)	ATC cells, xenograft	Shikonin	↓ GPX4, ↓ TXNRD1, ↓ PKM2, ↓ GLUT1, ↑ ROS	Dual inhibition of glycolysis and ferroptosis induction, tumor growth inhibition	[60]
Zhang et al. (2025)	ATC cells	EIF3H knockdown	β-catenin destabilization, ↓ Wnt/β-catenin signaling	Reduced proliferation, invasion, and ferroptosis resistance	[80]
Jin et al. (2024)	ATC cells	RON inhibition	MAPK/CREB blockade, ↓ GLUT1, ↓ HK2, ↓ PKM2, ↑ ferroptosis	Suppressed glycolysis, increased chemosensitivity	[43]
Lin et al. (2024)	ATC cells, xenograft	Isobavachalcone + doxorubicin	↑ ROS, ↑ MDA, ↑ iron, ↓ GSH, ↓ GPX4, ↓ SLC7A11	Synergistic ferroptosis activation, enhanced tumor suppression	[52]

↓, decreased; ↑, increased.

**Table 2:** Therapeutic strategies targeting ferroptosis in ATC.

Strategy	Mechanism	Representative Agents	Translational Implications
Initiators of ferroptosis	Promote iron overload, PUFA lipid peroxidation	Vitamin C, TDH, neferine, curcumin, shikonin	Direct tumor suppression; redox and metabolic dual targeting
GPX4 inhibition	Block lipid peroxide detoxification	RSL3, ML210	Strong ferroptosis induction, but toxicity risk
System Xc^−^ inhibition	Deplete cystine and GSH	Erastin, sulfasalazine	Synergistic with SIRT6, drug repurposing option
FSP1–CoQ_10_ inhibition	Block radical-trapping antioxidant system	iFSP1 (preclinical)	Synergy with GPX4 inhibitors, not tested in ATC
DHODH inhibition	Block mitochondrial lipid antioxidant defense	Brequinar (preclinical)	Potential in high mitochondrial activity ATC
Nrf2/HO-1 inhibition	Reduce transcriptional antioxidant defense	ML385, ZnPP (preclinical)	Overcome ferroptosis resistance in ATC
Wnt/β-catenin axis inhibition	Destabilize β-catenin, reduce ferroptosis resistance	EIF3H knockdown (preclinical)	Epitranscriptomic regulation of ferroptosis, novel biomarker potential
RTK/glycolysis inhibition	↓ MAPK/CREB signaling, suppress glycolysis, promote ferroptosis	RON inhibition (preclinical)	Cross-talk between metabolic rewiring and ferroptosis; enhances chemosensitivity
Combination regimens	Target oncogenic drivers + ferroptosis	Dabrafenib + RSL3, anlotinib + autophagy inhibitors, IBC + doxorubicin	Overcome kinase inhibitor or chemotherapy resistance, enhanced tumor suppression
Drug repurposing	Leverage approved drugs	Sulfasalazine, statins, artesunate	Accelerate translation into clinical testing
Nanoplatforms	Targeted delivery, multimodal therapy	FCIPL	Tumor-selective ferroptosis with imaging capacity

↓, decreased.

## Data Availability

No new data were created or analyzed in this study.

## Abbreviations

ACSL3, acyl-CoA synthetase long-chain family member 3; ACSL4, acyl-CoA synthetase long-chain family member 4; ATC, anaplastic thyroid cancer; BH_4_, tetrahydrobiopterin; BRAF^V600E^, B-Raf proto-oncogene serine/threonine kinase with valine-to-glutamic acid substitution at codon 600; CD71, transferrin receptor 1; CoQ_10_, coenzyme Q_10_; DHODH, dihydroorotate dehydrogenase; EIF3H, eukaryotic translation initiation factor 3 subunit H; EMT, epithelial-to-mesenchymal transition; FCIPL, Fe^3+^Cur-PFP@IR780-LIP nanoplatform; FSP1, ferroptosis suppressor protein 1; GPX4, glutathione peroxidase 4; GSH, glutathione; GPR34, G-protein coupled receptor 34; HO-1, heme oxygenase-1; IBC, isobavachalcone; IKE, imidazole ketone erastin; LPCAT3, lysophosphatidylcholine acyltransferase 3; MUFA, monounsaturated fatty acid; NCOA4, nuclear receptor coactivator 4; Nrf2, nuclear factor erythroid 2-related factor 2; PIK3CA, phosphatidylinositol-4,5-bisphosphate 3-kinase catalytic subunit alpha; PKM2, pyruvate kinase M2; PUFA, polyunsaturated fatty acid; RON, recepteur d’origine nantais (MST1R) receptor tyrosine kinase; ROS, reactive oxygen species; SIRT6, sirtuin 6; SLC7A11, solute carrier family 7 member 11; SWI/SNF, switch/sucrose non-fermentable chromatin remodeling complex; TDH, tenacissoside H; TFRC, transferrin receptor; TKI, tyrosine kinase inhibitor; TXNRD1, thioredoxin reductase 1; USP8, ubiquitin-specific protease 8.

## References

[1] Hamidi S., Maniakas A., Cabanillas M.E. (2025). Advances in Anaplastic Thyroid Cancer Treatment. Endocrinol. Metab. Clin. N. Am..

[2] Cleere E.F., Prunty S., O’Neill J.P. (2024). Anaplastic thyroid cancer: Improved understanding of what remains a deadly disease. Surgeon.

[3] Bible K.C., Kebebew E., Brierley J., Brito J.P., Cabanillas M.E., Clark T.J., Di Cristofano A., Foote R., Giordano T., Kasperbauer J. (2021). 2021 American Thyroid Association Guidelines for Management of Patients with Anaplastic Thyroid Cancer. Thyroid.

[4] Dixon S.J., Lemberg K.M., Lamprecht M.R., Skouta R., Zaitsev E.M., Gleason C.E., Patel D.N., Bauer A.J., Cantley A.M., Yang W.S. (2012). Ferroptosis: An iron-dependent form of nonapoptotic cell death. Cell.

[5] Stockwell B.R., Friedmann Angeli J.P., Bayir H., Bush A.I., Conrad M., Dixon S.J., Fulda S., Gascón S., Hatzios S.K., Kagan V.E. (2017). Ferroptosis: A Regulated Cell Death Nexus Linking Metabolism, Redox Biology, and Disease. Cell.

[6] Xie Y., Hou W., Song X., Yu Y., Huang J., Sun X., Kang R., Tang D. (2016). Ferroptosis: Process and function. Cell Death Differ..

[7] Alves F., Lane D., Nguyen T.P.M., Bush A.I., Ayton S. (2025). In defence of ferroptosis. Signal Transduct. Target. Ther..

[8] Pozdeyev N., Rose M.M., Bowles D.W., Schweppe R.E. (2020). Molecular therapeutics for anaplastic thyroid cancer. Semin. Cancer Biol..

[9] Silver Karcioglu A., Iwata A.J., Pusztaszeri M., Abdelhamid Ahmed A.H., Randolph G.W. (2022). The American Thyroid Association (ATA) integrates molecular testing into its framework for managing patients with anaplastic thyroid carcinoma (ATC): Update on the 2021 ATA ATC guidelines. Cancer Cytopathol..

[10] Liu X., Wang L., Xi X., Zhou T., Sun Z., Zhang B. (2025). Targeting ferroptosis: A novel insight into thyroid cancer therapy. Front. Endocrinol..

[11] Tian W., Su X., Hu C., Chen D., Li P. (2025). Ferroptosis in thyroid cancer: Mechanisms, current status, and treatment. Front. Oncol..

[12] Wu J., Liang J., Liu R., Lv T., Fu K., Jiang L., Ma W., Pan Y., Tan Z., Liu Q. (2023). Autophagic blockade potentiates anlotinib-mediated ferroptosis in anaplastic thyroid cancer. Endocr. Relat. Cancer.

[13] Wang X., Xu S., Zhang L., Cheng X., Yu H., Bao J., Lu R. (2021). Vitamin C induces ferroptosis in anaplastic thyroid cancer cells by ferritinophagy activation. Biochem. Biophys. Res. Commun..

[14] Li S., Zhang Y., Zhang J., Yu B., Wang W., Jia B., Chang J., Liu J. (2022). Neferine Exerts Ferroptosis-Inducing Effect and Antitumor Effect on Thyroid Cancer through Nrf2/HO-1/NQO1 Inhibition. J. Oncol..

[15] Dong P., Chi Y.B., Teng D.K., Lin Y.Q., Zhu L.Y., Li H.Q., Yang J.Y., Du J.R., Zhang Z.T., Ran H.T. (2025). Cascade-penetrating domino-ferroptosis nano inducer synergizes with sonodynamic therapy for anaplastic thyroid cancer. Mater. Today Bio.

[16] Lei G., Mao C., Yan Y., Zhuang L., Gan B. (2021). Ferroptosis, radiotherapy, and combination therapeutic strategies. Protein Cell.

[17] Zhou Q., Meng Y., Li D., Yao L., Le J., Liu Y., Sun Y., Zeng F., Chen X., Deng G. (2024). Ferroptosis in cancer: From molecular mechanisms to therapeutic strategies. Signal Transduct. Target. Ther..

[18] Diao J., Jia Y., Dai E., Liu J., Kang R., Tang D., Han L., Zhong Y., Meng L. (2024). Ferroptotic therapy in cancer: Benefits, side effects, and risks. Mol. Cancer.

[19] Karimi A.H., Zeng P.Y., Cecchini M., Barrett J.W., Pan H., Ying S., Le N., Mymryk J.S., Ailles L.E., Nichols A.C. (2025). NOVEL INSIGHTS IN ADVANCED THYROID CARCINOMA: FROM MECHANISMS TO TREATMENTS: Molecular insights into the origin, biology, and treatment of anaplastic thyroid carcinoma. Eur. Thyroid. J..

[20] Zou Z., Zhong L. (2025). Anaplastic thyroid cancer: Genetic roles, targeted therapy, and immunotherapy. Genes Dis..

[21] Jiang L., Kon N., Li T., Wang S.J., Su T., Hibshoosh H., Baer R., Gu W. (2015). Ferroptosis as a p53-mediated activity during tumour suppression. Nature.

[22] Xie Y., Zhu S., Song X., Sun X., Fan Y., Liu J., Zhong M., Yuan H., Zhang L., Billiar T.R. (2017). The Tumor Suppressor p53 Limits Ferroptosis by Blocking DPP4 Activity. Cell Rep..

[23] Hong Y., Ren T., Wang X., Liu X., Fei Y., Meng S., Han X., Sun C., Shen H., Li L. (2022). APR-246 triggers ferritinophagy and ferroptosis of diffuse large B-cell lymphoma cells with distinct TP53 mutations. Leukemia.

[24] Liu Y., Gu W. (2022). p53 in ferroptosis regulation: The new weapon for the old guardian. Cell Death Differ..

[25] Yin L., Luo X., Zhang X., Cheng B. (2024). The evolving process of ferroptosis in thyroid cancer: Novel mechanisms and opportunities. J. Cell. Mol. Med..

[26] Tarangelo A., Magtanong L., Bieging-Rolett K.T., Li Y., Ye J., Attardi L.D., Dixon S.J. (2018). p53 Suppresses Metabolic Stress-Induced Ferroptosis in Cancer Cells. Cell Rep..

[27] Andreani C., Bartolacci C., Scaglioni P.P. (2022). Ferroptosis: A Specific Vulnerability of RAS-Driven Cancers?. Front. Oncol..

[28] Hu J., Ghosh C., Khaket T.P., Yang Z., Tabdili Y., Alamaw E.D., Boufraqech M., Dixon S.J., Kebebew E. (2025). Dual Targeting of BRAF (V600E) and Ferroptosis Results in Synergistic Anticancer Activity via Iron Overload and Enhanced Oxidative Stress. bioRxiv.

[29] Yi J., Zhu J., Wu J., Thompson C.B., Jiang X. (2020). Oncogenic activation of PI3K-AKT-mTOR signaling suppresses ferroptosis via SREBP-mediated lipogenesis. Proc. Natl. Acad. Sci. USA.

[30] Zhang J., Zheng S., Xie R., Zhang J., Chen X., Xu S. (2025). Epigenetic control in thyroid cancer: Mechanisms and clinical perspective. Cell Death Discov..

[31] Magro G., Cataldo I., Amico P., Torrisi A., Vecchio G.M., Parenti R., Asioli S., Recupero D., D’Agata V., Mucignat M.T. (2011). Aberrant expression of TfR1/CD71 in thyroid carcinomas identifies a novel potential diagnostic marker and therapeutic target. Thyroid.

[32] Campisi A., Bonfanti R., Raciti G., Bonaventura G., Legnani L., Magro G., Pennisi M., Russo G., Chiacchio M.A., Pappalardo F. (2020). Gene Silencing of Transferrin-1 Receptor as a Potential Therapeutic Target for Human Follicular and Anaplastic Thyroid Cancer. Mol. Ther. Oncolytics.

[33] He J., Abikoye A.M., McLaughlin B.P., Middleton R.S., Sheldon R., Jones R.G., Schafer Z.T. (2023). Reprogramming of iron metabolism confers ferroptosis resistance in ECM-detached cells. iScience.

[34] Gao M., Monian P., Pan Q., Zhang W., Xiang J., Jiang X. (2016). Ferroptosis is an autophagic cell death process. Cell Res..

[35] Hou W., Xie Y., Song X., Sun X., Lotze M.T., Zeh H.J., Kang R., Tang D. (2016). Autophagy promotes ferroptosis by degradation of ferritin. Autophagy.

[36] Yang Z., Huang R., Wang Y., Guan Q., Li D., Wu Y., Liao T., Wang Y., Xiang J. (2023). SIRT6 drives sensitivity to ferroptosis in anaplastic thyroid cancer through NCOA4-dependent autophagy. Am. J. Cancer Res..

[37] Alonso E.G., Mascaró M., Schweitzer K., Giorgi G., Carballido J.A., Ibarra A., Clemente V., Pichel P., Recio S., Fernández Chávez L. (2025). Heme-oxygenase-1: A key player in thyroid carcinoma development. Endocr. Relat. Cancer.

[38] Consoli V., Sorrenti V., Grosso S., Vanella L. (2021). Heme Oxygenase-1 Signaling and Redox Homeostasis in Physiopathological Conditions. Biomolecules.

[39] Chen H., Li Z., Xu J., Zhang N., Chen J., Wang G., Zhao Y. (2023). Curcumin Induces Ferroptosis in Follicular Thyroid Cancer by Upregulating HO-1 Expression. Oxid. Med. Cell. Longev..

[40] Yang W.S., Kim K.J., Gaschler M.M., Patel M., Shchepinov M.S., Stockwell B.R. (2016). Peroxidation of polyunsaturated fatty acids by lipoxygenases drives ferroptosis. Proc. Natl. Acad. Sci. USA.

[41] Doll S., Proneth B., Tyurina Y.Y., Panzilius E., Kobayashi S., Ingold I., Irmler M., Beckers J., Aichler M., Walch A. (2017). ACSL4 dictates ferroptosis sensitivity by shaping cellular lipid composition. Nat. Chem. Biol..

[42] Sokol K.H., Lee C.J., Rogers T.J., Waldhart A., Ellis A.E., Madireddy S., Daniels S.R., House R.R.J., Ye X., Olesnavich M. (2025). Lipid availability influences ferroptosis sensitivity in cancer cells by regulating polyunsaturated fatty acid trafficking. Cell Chem. Biol..

[43] Jin X., Zhu H., Chen X., Yang Y., Song D. (2024). RON receptor tyrosine kinase regulates glycolysis through MAPK/CREB signaling to affect ferroptosis and chemotherapy sensitivity of thyroid cancer cells. Mol. Med. Rep..

[44] Magtanong L., Ko P.J., To M., Cao J.Y., Forcina G.C., Tarangelo A., Ward C.C., Cho K., Patti G.J., Nomura D.K. (2019). Exogenous Monounsaturated Fatty Acids Promote a Ferroptosis-Resistant Cell State. Cell Chem. Biol..

[45] Cao Y., Li J., Chen Y., Wang Y., Liu Z., Huang L., Liu B., Feng Y., Yao S., Zhou L. (2025). Monounsaturated fatty acids promote cancer radioresistance by inhibiting ferroptosis through ACSL3. Cell Death Dis..

[46] Cui W., Liu D., Gu W., Chu B. (2021). Peroxisome-driven ether-linked phospholipids biosynthesis is essential for ferroptosis. Cell Death Differ..

[47] von Roemeling C.A., Copland J.A. (2016). Targeting lipid metabolism for the treatment of anaplastic thyroid carcinoma. Expert Opin. Ther. Targets.

[48] Delmas D., Mialhe A., Cotte A.K., Connat J.L., Bouyer F., Hermetet F., Aires V. (2025). Lipid metabolism in cancer: Exploring phospholipids as potential biomarkers. Biomed. Pharmacother..

[49] Guo W., Duan Z., Wu J., Zhou B.P. (2025). Epithelial-mesenchymal transition promotes metabolic reprogramming to suppress ferroptosis. Semin. Cancer Biol..

[50] Dang T., Yu J., Yu Y., Jiang J., Shi Y., Yu S., Peng C., Min X., Xiong Y., Long P. (2024). GPX4 inhibits apoptosis of thyroid cancer cells through regulating the FKBP8/Bcl-2 axis. Cancer Biomark..

[51] Sekhar K.R., Hanna D.N., Cyr S., Baechle J.J., Kuravi S., Balusu R., Rathmell K., Baregamian N. (2022). Glutathione peroxidase 4 inhibition induces ferroptosis and mTOR pathway suppression in thyroid cancer. Sci. Rep..

[52] Lin S., Cai H., Song X. (2024). Synergy between isobavachalcone and doxorubicin suppressed the progression of anaplastic thyroid cancer through ferroptosis activation. Braz. J. Med. Biol. Res..

[53] D’Aprile S., Denaro S., Pavone A.M., Giallongo S., Giallongo C., Distefano A., Salvatorelli L., Torrisi F., Giuffrida R., Forte S. (2023). Anaplastic thyroid cancer cells reduce CD71 levels to increase iron overload tolerance. J. Transl. Med..

[54] Bersuker K., Hendricks J.M., Li Z., Magtanong L., Ford B., Tang P.H., Roberts M.A., Tong B., Maimone T.J., Zoncu R. (2019). The CoQ oxidoreductase FSP1 acts parallel to GPX4 to inhibit ferroptosis. Nature.

[55] Doll S., Freitas F.P., Shah R., Aldrovandi M., da Silva M.C., Ingold I., Goya Grocin A., Xavier da Silva T.N., Panzilius E., Scheel C.H. (2019). FSP1 is a glutathione-independent ferroptosis suppressor. Nature.

[56] Mao C., Liu X., Zhang Y., Lei G., Yan Y., Lee H., Koppula P., Wu S., Zhuang L., Fang B. (2021). DHODH-mediated ferroptosis defence is a targetable vulnerability in cancer. Nature.

[57] Kraft V.A.N., Bezjian C.T., Pfeiffer S., Ringelstetter L., Müller C., Zandkarimi F., Merl-Pham J., Bao X., Anastasov N., Kössl J. (2020). GTP Cyclohydrolase 1/Tetrahydrobiopterin Counteract Ferroptosis through Lipid Remodeling. ACS Cent. Sci..

[58] Trotter E.W., Grant C.M. (2003). Non-reciprocal regulation of the redox state of the glutathione-glutaredoxin and thioredoxin systems. EMBO Rep..

[59] de Cubas L., Boronat S., Vega M., Domènech A., Gómez-Armengol F., Artemov A., Lyublinskaya O., Ayté J., Hidalgo E. (2025). The glutathione system maintains the thiol redox balance in the mitochondria of fission yeast. Free Radic. Biol. Med..

[60] Yang C., Yang L., Li D., Tan J., Jia Q., Sun H., Meng Z., Wang Y. (2024). Shikonin inhibits the growth of anaplastic thyroid carcinoma cells by promoting ferroptosis and inhibiting glycolysis. Heliyon.

[61] Anandhan A., Dodson M., Shakya A., Chen J., Liu P., Wei Y., Tan H., Wang Q., Jiang Z., Yang K. (2023). NRF2 controls iron homeostasis and ferroptosis through HERC2 and VAMP8. Sci. Adv..

[62] Tang D., Kang R. (2024). NFE2L2 and ferroptosis resistance in cancer therapy. Cancer Drug Resist..

[63] Anandhan A., Dodson M., Schmidlin C.J., Liu P., Zhang D.D. (2020). Breakdown of an Ironclad Defense System: The Critical Role of NRF2 in Mediating Ferroptosis. Cell Chem. Biol..

[64] Lee J., Seo Y., Roh J.L. (2025). Ferroptosis and Nrf2 Signaling in Head and Neck Cancer: Resistance Mechanisms and Therapeutic Prospects. Antioxidants.

[65] Renaud C.O., Ziros P.G., Chartoumpekis D.V., Bongiovanni M., Sykiotis G.P. (2019). Keap1/Nrf2 Signaling: A New Player in Thyroid Pathophysiology and Thyroid Cancer. Front. Endocrinol..

[66] Gong Z., Xue L., Wei M., Liu Z., Vlantis A.C., van Hasselt C.A., Chan J.Y.K., Li D., Zeng X., Tong M.C.F. (2021). The Knockdown of Nrf2 Suppressed Tumor Growth and Increased the Sensitivity to Lenvatinib in Anaplastic Thyroid Cancer. Oxid. Med. Cell. Longev..

[67] Xu Y., Hu S., Chen R., Xu S., Yu G., Ji L. (2025). Interplay between Nrf2 and ROS in regulating epithelial-mesenchymal transition: Implications for cancer metastasis and therapy. Mol. Biol. Rep..

[68] Luo H., Xia X., Kim G.D., Liu Y., Xue Z., Zhang L., Shu Y., Yang T., Chen Y., Zhang S. (2021). Characterizing dedifferentiation of thyroid cancer by integrated analysis. Sci. Adv..

[69] Lu L., Wang J.R., Henderson Y.C., Bai S., Yang J., Hu M., Shiau C.K., Pan T., Yan Y., Tran T.M. (2023). Anaplastic transformation in thyroid cancer revealed by single-cell transcriptomics. J. Clin. Investig..

[70] Luo J., Wang Y., Zhao L., Wang C., Zhang Z. (2022). Anti-Anaplastic Thyroid Cancer (ATC) Effects and Mechanisms of PLX3397 (Pexidartinib), a Multi-Targeted Tyrosine Kinase Inhibitor (TKI). Cancers.

[71] Liao T., Zeng Y., Xu W., Shi X., Shen C., Du Y., Zhang M., Zhang Y., Li L., Ding P. (2025). A spatially resolved transcriptome landscape during thyroid cancer progression. Cell Rep. Med..

[72] Wan Y., Li G., Cui G., Duan S., Chang S. (2025). Reprogramming of Thyroid Cancer Metabolism: From Mechanism to Therapeutic Strategy. Mol. Cancer.

[73] Chen Y., Pan G., Wu F., Zhang Y., Li Y., Luo D. (2024). Ferroptosis in thyroid cancer: Potential mechanisms, effective therapeutic targets and predictive biomarker. Biomed. Pharmacother..

[74] Hangauer M.J., Viswanathan V.S., Ryan M.J., Bole D., Eaton J.K., Matov A., Galeas J., Dhruv H.D., Berens M.E., Schreiber S.L. (2017). Drug-tolerant persister cancer cells are vulnerable to GPX4 inhibition. Nature.

[75] Wu J., Minikes A.M., Gao M., Bian H., Li Y., Stockwell B.R., Chen Z.N., Jiang X. (2019). Intercellular interaction dictates cancer cell ferroptosis via NF2-YAP signalling. Nature.

[76] He W., Hu X., Ge M., Meng K. (2025). The central role of ferroptosis-induced therapy mediated by tenacissoside H in anaplastic thyroid cancer. J. Ethnopharmacol..

[77] Noronha S., Liu Y., Geneti G., Li H., Wu X., Sun D., Gujar V., Furusawa T., Lobanov A., Cam M. (2025). CRISPR-Based Gene Dependency Screens reveal Mechanism of BRAF Inhibitor Resistance in Anaplastic Thyroid Cancer. bioRxiv.

[78] Guo Y., Liang J., Ding L., Wu J., Teng W., Wang J., Jiang L., Tan Z. (2025). The endoplasmic reticulum stress-ferroptosis reciprocal signaling orchestrates anti-tumor effect of anlotinib in anaplastic thyroid cancer. Cancer Cell Int..

[79] Yan B., Guo J., Huang M., Li Z., Sun J., Tan H., Lai W., Chang S. (2025). GPR34 Stabilized by Deubiquitinase USP8 Suppresses Ferroptosis of ATC. Mediat. Inflamm..

[80] Zhang Z., Zhou D., Qiu X., Xia F., Li X. (2025). N6-methyladenosine-mediated EIF3H promotes anaplastic thyroid cancer progression and ferroptosis resistance by stabilizing β-catenin. Free Radic. Biol. Med..

[81] Hamidi S., Dadu R., Zafereo M.E., Ferrarotto R., Wang J.R., Maniakas A., Gunn G.B., Lee A., Spiotto M.T., Iyer P.C. (2024). Initial Management of BRAF V600E-Variant Anaplastic Thyroid Cancer: The FAST Multidisciplinary Group Consensus Statement. JAMA Oncol..

[82] Subbiah V., Kreitman R.J., Wainberg Z.A., Cho J.Y., Schellens J.H.M., Soria J.C., Wen P.Y., Zielinski C.C., Cabanillas M.E., Boran A. (2022). Dabrafenib plus trametinib in patients with BRAF V600E-mutant anaplastic thyroid cancer: Updated analysis from the phase II ROAR basket study. Ann. Oncol..

[83] Wang J., Wu N., Peng M., Oyang L., Jiang X., Peng Q., Zhou Y., He Z., Liao Q. (2023). Ferritinophagy: Research advance and clinical significance in cancers. Cell Death Discov..

[84] Kuwata H., Hara S. (2019). Role of acyl-CoA synthetase ACSL4 in arachidonic acid metabolism. Prostaglandins Other Lipid Mediat..

[85] Kim J.W., Lee J.Y., Oh M., Lee E.W. (2023). An integrated view of lipid metabolism in ferroptosis revisited via lipidomic analysis. Exp. Mol. Med..

[86] Ping P., Ma Y., Xu X., Li J. (2025). Reprogramming of fatty acid metabolism in thyroid cancer: Potential targets and mechanisms. Chin. J. Cancer Res..

[87] Wood W.M., Sharma V., Bauerle K.T., Pike L.A., Zhou Q., Fretwell D.L., Schweppe R.E., Haugen B.R. (2011). PPARγ Promotes Growth and Invasion of Thyroid Cancer Cells. PPAR Res..

[88] Pratama A.M., Sharma M., Naidu S., Bömmel H., Prabhuswamimath S.C., Madhusudhan T., Wihadmadyatami H., Bachhuka A., Karnati S. (2024). Peroxisomes and PPARs: Emerging role as master regulators of cancer metabolism. Mol. Metab..

[89] Hendricks J.M., Doubravsky C.E., Wehri E., Li Z., Roberts M.A., Deol K.K., Lange M., Lasheras-Otero I., Momper J.D., Dixon S.J. (2023). Identification of structurally diverse FSP1 inhibitors that sensitize cancer cells to ferroptosis. Cell Chem. Biol..

[90] Cao J., Chen X., Chen L., Lu Y., Wu Y., Deng A., Pan F., Huang H., Liu Y., Li Y. (2025). DHODH-mediated mitochondrial redox homeostasis: A novel ferroptosis regulator and promising therapeutic target. Redox Biol..

[91] Roh J.L., Kim E.H., Jang H., Shin D. (2017). Nrf2 inhibition reverses the resistance of cisplatin-resistant head and neck cancer cells to artesunate-induced ferroptosis. Redox Biol..

[92] Shin D., Kim E.H., Lee J., Roh J.L. (2018). Nrf2 inhibition reverses resistance to GPX4 inhibitor-induced ferroptosis in head and neck cancer. Free Radic. Biol. Med..

[93] Zhang C., Liu X., Jin S., Chen Y., Guo R. (2022). Ferroptosis in cancer therapy: A novel approach to reversing drug resistance. Mol. Cancer.

[94] Bagheri-Yarmand R., Busaidy N.L., McBeath E., Danysh B.P., Evans K.W., Moss T.J., Akcakanat A., Ng P.K.S., Knippler C.M., Golden J.A. (2021). RAC1 Alterations Induce Acquired Dabrafenib Resistance in Association with Anaplastic Transformation in a Papillary Thyroid Cancer Patient. Cancers.

[95] Kiyota N., Koyama T., Sugitani I. (2025). Anticancer drug therapy for anaplastic thyroid cancer. Eur. Thyroid J..

[96] Sekihara K., Himuro H., Toda S., Saito N., Hirayama R., Suganuma N., Sasada T., Hoshino D. (2024). Recent Trends and Potential of Radiotherapy in the Treatment of Anaplastic Thyroid Cancer. Biomedicines.

[97] Gao W., Wang X., Zhou Y., Wang X., Yu Y. (2022). Autophagy, ferroptosis, pyroptosis, and necroptosis in tumor immunotherapy. Signal Transduct. Target. Ther..

[98] Ebrahimnezhad M., Valizadeh A., Yousefi B. (2025). Ferroptosis and immunotherapy: Breaking barriers in cancer treatment resistance. Crit. Rev. Oncol. Hematol..

[99] Wang W., Green M., Choi J.E., Gijón M., Kennedy P.D., Johnson J.K., Liao P., Lang X., Kryczek I., Sell A. (2019). CD8(+) T cells regulate tumour ferroptosis during cancer immunotherapy. Nature.

[100] Han P.Z., Ye W.D., Yu P.C., Tan L.C., Shi X., Chen X.F., He C., Hu J.Q., Wei W.J., Lu Z.W. (2024). A distinct tumor microenvironment makes anaplastic thyroid cancer more lethal but immunotherapy sensitive than papillary thyroid cancer. JCI Insight.

[101] Jiang W., Hu J.W., He X.R., Jin W.L., He X.Y. (2021). Statins: A repurposed drug to fight cancer. J. Exp. Clin. Cancer Res..

[102] Huang Q.F., Li Y.H., Huang Z.J., Jun M., Wang W., Chen X.L., Wang G.H. (2023). Artesunate carriers induced ferroptosis to overcome biological barriers for anti-cancer. Eur. J. Pharm. Biopharm..

[103] Chen X., Kang R., Kroemer G., Tang D. (2021). Broadening horizons: The role of ferroptosis in cancer. Nat. Rev. Clin. Oncol..

[104] Ma W., Hu N., Xu W., Zhao L., Tian C., Kamei K.I. (2024). Ferroptosis inducers: A new frontier in cancer therapy. Bioorganic Chem..

[105] Luo Y., Bai X.Y., Zhang L., Hu Q.Q., Zhang N., Cheng J.Z., Hou M.Z., Liu X.L. (2024). Ferroptosis in Cancer Therapy: Mechanisms, Small Molecule Inducers, and Novel Approaches. Drug Des. Dev. Ther..

[106] Chen X., Zhang T., Su W., Dou Z., Zhao D., Jin X., Lei H., Wang J., Xie X., Cheng B. (2022). Mutant p53 in cancer: From molecular mechanism to therapeutic modulation. Cell Death Dis..

[107] Pakkianathan J., Yamauchi C.R., Barseghyan L., Cruz J., Simental A.A., Khan S. (2025). Mutational Landmarks in Anaplastic Thyroid Cancer: A Perspective of a New Treatment Strategy. J. Clin. Med..

[108] Chen H., Peng F., Xu J., Wang G., Zhao Y. (2023). Increased expression of GPX4 promotes the tumorigenesis of thyroid cancer by inhibiting ferroptosis and predicts poor clinical outcomes. Aging.

[109] Lee J., Roh J.L. (2022). SLC7A11 as a Gateway of Metabolic Perturbation and Ferroptosis Vulnerability in Cancer. Antioxidants.

[110] Yang Y., Wang Y., Guo L., Gao W., Tang T.L., Yan M. (2022). Interaction between macrophages and ferroptosis. Cell Death Dis..

[111] Shi L., Liu Y., Li M., Luo Z. (2022). Emerging roles of ferroptosis in the tumor immune landscape: From danger signals to anti-tumor immunity. Febs J..

[112] Wächter S., Bartsch D.K., Knorrenschild J.R., Pehl A., Eilsberger F., Pfestroff A., Luster M., Holzer K., Neubauer A., Maurer E. (2025). Mutation-based, neoadjuvant treatment for advanced anaplastic thyroid carcinoma. Front. Endocrinol..

